# Do ethnicity, degree of family relationship, and the spondyloarthritis subtype in affected relatives influence the association between a positive family history for spondyloarthritis and HLA-B27 carriership? Results from the worldwide ASAS cohort

**DOI:** 10.1186/s13075-018-1672-2

**Published:** 2018-08-03

**Authors:** Miranda van Lunteren, Alexandre Sepriano, Robert Landewé, Joachim Sieper, Martin Rudwaleit, Désirée van der Heijde, Floris van Gaalen

**Affiliations:** 1 Department of Rheumatology, Leiden University Medical Center, P.O. Box 9600, 2300 RC Leiden, the Netherlands; 2 NOVA Medical School, Universidade Nova de Lisboa, Lisbon, Portugal; 3 Department of Rheumatology, Amsterdam Rheumatology & Immunology Center, Amsterdam, the Netherlands; 4 Department of Rheumatology, Zuyderland Hospital, Heerlen, the Netherlands; 5 Department of Rheumatology, Charité Campus Benjamin Franklin, Berlin, Germany; 6 German Rheumatism Research Centre, Berlin, Germany; 7 Department of Internal Medicine and Rheumatology, Klinikum Bielefeld Rosenhöhe, Bielefeld, Germany

**Keywords:** Axial spondyloarthritis, HLA-B27, Positive family history, Ethnicity, Degree of family relationship

## Abstract

**Background:**

The Assessment of SpondyloArthritis international Society (ASAS) defines a positive family history (PFH) of spondyloarthritis (SpA) as the presence of ankylosing spondylitis (AS), acute anterior uveitis (AAU), reactive arthritis (ReA), inflammatory bowel disease (IBD), and/or psoriasis in first-degree relatives (FDR) or second-degree relatives (SDR). In two European cohorts, a PFH of AS and AAU, but not other subtypes, was associated with human leukocyte antigen B27 (HLA-B27) carriership in patients suspected of axial SpA (axSpA). Because the importance of ethnicity or degree of family relationship is unknown, we investigated the influence of ethnicity, FDR, or SDR on the association between a PFH and HLA-B27 carriership in patients suspected of axSpA.

**Methods:**

Baseline data from the ASAS cohort of patients suspected of axSpA were analyzed. Univariable analyses were performed. Each disease (AS, AAU, psoriasis, IBD, ReA) in a PFH according to the ASAS definition was a determinant in separate models with HLA-B27 carriership as outcome. Analyses were stratified for self-reported ethnicity, FDR, and SDR. Analyses were repeated in multivariable models to investigate independent associations.

**Results:**

A total of 594 patients were analyzed (mean [SD] age 33.7 [11.7] years; 46% male; 52% HLA-B27+; 59% white, 36% Asian, 5% other). A PFH was associated with HLA-B27 carriership in patients with a white (OR, 2.3, 95% CI, 1.4–3.9) or Asian ethnicity (OR, 3.1, 95% CI, 1.6–5.8) and with a PFH in FDR (OR, 2.9, 95% CI, 1.8–4.5), but not with a PFH in SDR (OR, 1.7, 95% CI, 0.7–3.8) or in other ethnicities. A PFH of AS was positively associated with HLA-B27 carriership in all subgroups (white OR, 7.1; 95% CI, 2.9–17.1; Asian OR, 5.7; 95% CI, 2.5–13.2; FDR OR, 7.8; 95% CI, 3.8–16.0; SDR OR, 3.7; 95% CI, 1.2–11.6). A PFH of AAU, ReA, IBD, or psoriasis was never positively associated with HLA-B27 carriership. In the multivariate analysis, similar results were found.

**Conclusions:**

In the ASAS cohort, a PFH of AS, but not of AAU, ReA, IBD, or psoriasis, was associated with HLA-B27 carriership regardless of white or Asian ethnicity or degree of family relationship. This cohort and two European cohorts show that a PFH of AS and possibly a PFH of AAU can be used to identify patients who are more likely to be HLA-B27-positive and therefore may have an increased risk of axSpA.

## Background

Axial spondyloarthritis (axSpA) is a chronic inflammatory disease that causes inflammation mainly in the sacroiliac joints (SI) and spine [1]. In patients with ankylosing spondylitis (AS), also termed radiographic axSpA, susceptibility is thought to be largely genetically determined, and the strongest known genetic risk factor for axSpA is human leukocyte antigen B27 (HLA-B27) [1, 2]. Different prevalence rates of axSpA are reported across geographical regions, which have been related to the varying prevalence of HLA-B27 worldwide [3]. A family history of spondyloarthritis (SpA) is common in patients with AS [4], and HLA-B27-positive first-degree relatives of HLA-B27-positive patients with AS are 16 times more likely to develop AS than HLA-B27-positive individuals in the general population [5]. Additionally, several studies have shown that first-degree relatives of a patient with AS have a higher risk of developing AS than second-degree relatives [6–8]. Therefore, a positive family history (PFH) of SpA, and in particular a PFH in first-degree relatives of patients with SpA, is thought to be a risk factor of axSpA in patients with chronic back pain (CBP), and a PFH of SpA is a component of several SpA classification criteria [9, 10].

The Assessment of SpondyloArthritis international Society (ASAS) defined, by consensus, a PFH of AS, acute anterior uveitis (AAU), reactive arthritis (ReA), inflammatory bowel disease (IBD), and/or psoriasis in first- or second-degree relatives as an SpA feature in the ASAS classification criteria for axSpA [11]. Because a PFH of SpA is thought to increase the risk for axSpA in patients with CBP, it has been incorporated into several referral strategies for patients with CBP suspected of axSpA [12, 13].

So far, only one study has investigated the performance of the definition of a PFH in identifying patients with an increased risk for axSpA. Ez-Zaitouni et al. reported that in two cohorts predominantly Caucasian CBP patients suspected of axSpA, a PFH for AS and AAU was positively associated with HLA-B27 carriership. A PFH for ReA, IBD, or psoriasis was not associated with HLA-B27 carriership and did not point to HLA-B27 carriership in patients with back pain [14]. Unfortunately, this study did not to distinguish between first- or second-degree relatives, and therefore it was unclear if a distinction in a PFH in first- or second-degree relatives matters. Moreover, because the study was performed in two European cohorts, it was unclear if the authors’ findings are relevant to populations outside Europe.

The ASAS cohort provides a unique opportunity to study the performance of the current ASAS definition of a PFH in patients of different ethnicities who have CBP and are suspected of axSpA [11, 15, 16]. Therefore, we investigated in this international cohort the impact of ethnicity and degree of family relationship on the association between the current ASAS definition of a PFH and the presence of HLA-B27 in patients suspected of axSpA.

## Methods

The ASAS cohort is an international cohort that includes patients with a suspicion of axSpA (> 3 months of back pain, age at onset < 45 years, with or without peripheral symptoms) or peripheral SpA (pSpA; current peripheral arthritis and/or dactylitis and/or enthesitis but without current CBP) [11, 15, 16]. Worldwide, 975 patients were included by 29 ASAS centers between November 2005 and January 2009. Patients were included in a consecutive manner, including all eligible patients or every first to third eligible patient per day. Local ethical committees approved the study, and informed consent was obtained from all study participants before inclusion [11, 15, 16].

### Data collection

At baseline, clinical, laboratory, and imaging data were collected from all patients, including HLA-B27 carriership and radiography of the SI. Magnetic resonance imaging of the SI (MRI-SI) was considered obligatory for the first 20 patients of each center. Patients were diagnosed by the treating rheumatologist, and for each patient, the level of confidence regarding the diagnosis on an 11-point numerical rating scale from 0 (not confident at all) to 10 (very confident) was provided. For the current analysis, only patients with a certain diagnosis of axSpA or no axSpA (confidence level ≥ 6) were analyzed.

In addition, patients were asked to report their ethnicity using an open-ended question. Self-reported ethnicities were white, Asian, black, East Indian, Hispanic/Latino, mixed, and Turkish. The self-reported ethnicities were reclassified into white, Asian, and other ethnicities (Hispanic/Latino *n* = 11, black *n* = 7, mixed *n* = 4, Turkish *n* = 3, East Indian *n* = 2, unknown = 5).

The ASAS expert definition was used to assess if patients had a PFH of SpA (ASAS PFH) (i.e., the presence of AS, AAU, psoriasis, IBD, and/or ReA in first- or second-degree relatives) [11]. Patients were asked to report if any family history disease was present in a relative and in which relative(s). In the ASAS expert definition, father, mother, sister, brother, daughter, and son are defined as first-degree relatives and grandmother, grandfather, aunt, uncle, niece, and nephew as second-degree relatives [11]. Furthermore, in addition to the ASAS definition, granddaughter, grandson, half-sister, and half-brother were also considered to be second-degree relatives.

### Data analysis

Baseline data were analyzed. Continuous variables were presented as mean and SD and categorical variables as frequencies (proportions). Univariable logistic models, stratified by self-reported ethnicity or degree of family relationship (a PFH in first- or only second-degree relatives), were used to assess the association between each disease (AS, AAU, ReA, IBD, psoriasis) in a PFH and HLA-B27 carriership. The analyses were repeated in multivariable logistic models to investigate if each family history disease was associated, independently of other PFH subtypes, with HLA-B27 carriership. Patients with a PFH in both first- and second-degree relatives were classified into the group of patients with PFH in a first-degree relative (*n* = 11) for both the univariable and multivariable analyses. STATA SE version 14 software (StataCorp, College Station, TX, USA) was used to perform data analyses.

## Results

In the ASAS cohort, 642 patients were diagnosed with a confidence level ≥ 6 as axSpA or no axSpA. Patients were excluded from the analysis if the family history was missing (*n* = 48). For the current analysis, 594 patients were used. Patients had a mean age (SD) of 33.7 (11.7) years, and 46% were male. Mean symptom duration was 7.1 (9.0) years, and a mean of 2.4 (1.6) SpA features, excluding HLA-B27 and imaging, were present. Of the sample, 52% were HLA-B27-positive, 20% had radiographic sacroiliitis, and 45% had active inflammation by MRI-SI (Table 1). Sixty-two percent of the patients were diagnosed as axSpA.

**Table 1:** Baseline characteristics of chronic back pain patients suspected of axial spondyloarthritis included in the ASAS cohort

Characteristics	Data (*N* = 594)
Age at baseline, years	33.7 (11.7)
Male sex	276 (46%)
Duration of back pain, years^a^	7.1 (9.0)
Self-reported ethnicity	
White	348 (59%)
Asian	214 (36%)
Other^b^	32 (5%)
IBP (according to experts’ definition)	301/532 (57%)
Good response to NSAIDs^c^	274 (46%)
Peripheral arthritis^d^	197 (33%)
Enthesitis^d^	241 (41%)
AAU^d^	53 (9%)
Dactylitis^d^	23 (4%)
Psoriasis^d^	22 (4%)
IBD^d^	8 (1%)
Positive family history according to ASAS definition	135 (23%)
Positive family history of AS	87 (15%)
Positive family history of AAU	7 (1%)
Positive family history of ReA	8 (1%)
Positive family history of IBD	12 (2%)
Positive family history of Psoriasis	36 (6%)
Total number of SpA-related diseases in first- or second-degree relatives	
Number of patients with one disease	120 (20%)
Number of patients with two diseases	15 (3%)
Total number of family members with SpA-related diseases	
Number of patients with one relative	102 (17%)
Number of patients with two relatives	30 (5%)
Number of patients with three relatives	3 (1%)
Total number of patients with positive family history in	
First-degree relatives only	100 (17%)
Second-degree relatives only	25 (4%)
Both first- and second-degree relatives	10 (2%)
HLA-B27 positivity	310 (52%)
Elevated CRP/ESR	185 (31%)
Definite radiographic sacroiliitis^e^	119/593 (20%)
Presence of active inflammation on MRI-SI	189/424 (45%)
Number of SpA features^f,g^	2.4 (1.6)
Clinical diagnosis of axSpA^h^	368 (62%)

*Abbreviations: AAU* Acute anterior uveitis, *AS* Ankylosing spondylitis, *ASAS* Assessment of SpondyloArthritis international Society, *(ax)SpA* (Axial) spondyloarthritis, *CRP* C-reactive protein, *ESR* Erythrocyte sedimentation rate, *HLA-B27* Human leukocyte antigen B27, *IBD* Inflammatory bowel disease, *IBP* Inflammatory back pain, *MRI-SI* Magnetic resonance imaging of the sacroiliac joints, *NSAID* Nonsteroidal anti-inflammatory drug, *ReA* Reactive arthritis, *SI* Sacroiliac joint

Results are presented as mean ± SD unless specified otherwise

^a^ < 5% missing values

^b^ Self-reported ethnicity was missing for five patients, who are included in this category, and other self-reported ethnicities are black, East Indian, Hispanic/Latino, mixed, or Turkish

^c^ Back pain not present anymore or is much better 24–48 hours after a full dose of NSAID

^d^ Past or present condition

^e^ Grade ≥ 2 bilateral or grade ≥ 3 unilateral

^f^ Excluding HLA-B27 carriership and imaging

^g^ < 20% missing values

^h^ Level of confidence regarding the diagnosis is ≥ 6

In total, 59% of patients reported to be white, 36% to be Asian, and 5% reported another ethnicity (Table 1). An ASAS PFH was reported by 23% of the patients; a PFH of AS was the most frequently reported family history, with 15% (64% of all PFH), and a PFH of AAU was the least often reported, with 1% (5% of all PFH), among all patients (Table 1). An ASAS PFH in first-degree relatives only was reported in 17% of patients, in second-degree relatives in 4%, and in both first- and second-degree relatives in 2% of the patients.

An ASAS PFH and a PFH of AS were positively associated with HLA-B27 in all patients suspected of axSpA (ASAS PFH OR, 2.6; 95% CI, 1.7–3.9; PFH of AS OR, 6.5; 95% CI, 3.5–12.1). When these patients were stratified according to ethnicity and degree of family relationship, positive associations were found between an ASAS PFH and HLA-B27 carriership in patients with a self-reported white (OR, 2.3; 95% CI, 1.4–3.9) or Asian ethnicity (OR, 3.1; 95% CI, 1.6–5.8) and with a PFH in first-degree relatives (OR, 2.9; 95% CI, 1.8–4.5), but not in second-degree relatives (OR, 1.7; 95% CI, 0.7–3.8) (Table 2). A PFH of AS was positively associated with HLA-B27 carriership in all subgroups (white OR, 7.1; 95% CI, 2.9–17.1; Asian OR, 5.7; 95% CI, 2.5–13.2; first-degree relatives OR, 7.8; 95% CI, 3.8–16.0; second-degree relatives OR, 3.7; 95% CI, 1.2–11.6). A PFH of AAU, ReA, IBD, or psoriasis was not positively associated with HLA-B27 carriership in patients suspected of axSpA, regardless of the degree of family relationship or ethnicity (Table 2). In the multivariable analysis, similar results were found (data not shown).

**Table 2:** Univariable associations between a positive family history and HLA-B27 carriership in patients suspected of axial spondyloarthritis (*n* = 594)

	HLA-B27+ (*n* = 310)	HLA-B27− (*n* = 284)	OR (95% CI)	*p* Value
Positive family history according to ASAS definition				
Stratified by self-reported ethnicity				
White	**54**	**26**	**2.3 (1.4–3.9)**	**0.001**
Asian	**38**	**14**	**3.1 (1.6–5.8)**	**0.001**
Other ethnicities^a^	2	1	2.3 (0.2–25.0)	0.509
Stratified by degree of family relationship				
First-degree relatives	**79**	**31**	**2.9 (1.8–4.5)**	**< 0.001**
Only second-degree relatives	15	10	1.7 (0.7–3.8)	0.212
Positive family history of AS				
Stratified by self-reported ethnicity				
White	**37**	**6**	**7.1 (2.9–17.1)**	**< 0.001**
Asian	**35**	**7**	**5.7 (2.5–13.2)**	**< 0.001**
Other ethnicities^a^	2	0	n.a.	n.a.
Stratified by degree of family relationship				
First-degree relatives	**61**	**9**	**7.8 (3.8–16.0)**	**< 0.001**
Only second-degree relatives	**13**	**4**	**3.7 (1.2–11.6)**	**0.023**
Positive family history of AAU				
Stratified by self-reported ethnicity				
White	4	0	n.a.	n.a.
Asian	2	1	1.9 (0.2–20.7)	0.613
Other ethnicities^a^	0	0	n.a.	n.a.
Stratified by degree of family relationship				
First-degree relatives	5	1	4.7 (0.5–40.1)	0.162
Only second-degree relatives	1	0	n.a.	n.a.
Positive family history of ReA				
Stratified by self-reported ethnicity				
White	2	2	0.9 (0.1–6.5)	0.924
Asian	1	3	0.3 (0.03–2.9)	0.302
Other ethnicities^a^	0	0	n.a.	n.a.
Stratified by degree of family relationship				
First-degree relatives	3	2	1.4 (0.2–8.2)	0.735
Only second-degree relatives	0	3	n.a	n.a
Positive family history of IBD				
Stratified by self-reported ethnicity				
White	2	7	0.3 (0.05–1.2)	0.089
Asian	0	2	n.a.	n.a.
Other ethnicities^a^	0	1	n.a.	n.a.
Stratified by degree of family relationship				
First-degree relatives	1	7	0.1 (0.02–1.0)	0.054
Only second-degree relatives	1	3	0.3 (0.03–2.9)	0.294
Positive family history of psoriasis				
Stratified by self-reported ethnicity				
White	16	15	1.0 (0.5–2.0)	0.938
Asian	2	2	0.9 (0.1–6.5)	0.926
Other ethnicities^a^	0	1	n.a.	n.a.
Stratified by degree of family relationship				
First-degree relatives	15	14	1.0 (0.5–2.1)	0.949
Only second-degree relatives	3	4	0.7 (0.2–3.1)	0.620

Statistically significant results are printed in bold

*Abbreviations: AAU* Acute anterior uveitis, *AS* Ankylosing spondylitis, *HLA-B27* Human leukocyte antigen B27, *IBD* Inflammatory bowel disease, *n.a.* Not applicable, *ReA* Reactive arthritis

^a^Self-reported ethnicities was missing for five patients, who are included in this category, and other ethnicities are black, East Indian, Hispanic/Latino, mixed, or Turkish

## Discussion

In patients suspected of axSpA in the worldwide ASAS cohort, a PFH of AS was the predominant PFH subtype and was associated, independently of other PFH subtypes, with HLA-B27 carriership, regardless of self-reported ethnicity or degree of family relationship. No positive associations were found between a PFH of AAU, ReA, IBD, or psoriasis and HLA-B27 carriership. However, it should be noted that in patients with CBP suspected of axSpA, a PFH of AAU, ReA, and IBD was less common than a PFH of AS, which is in line with previous studies. Somewhat stronger associations were found for patients with white ethnicity and for patients with a PFH in first-degree relatives. Nonetheless, a PFH of AS was strongly associated with HLA-B27 carriership in both white and Asian patients and in both first- and second-degree relatives.

Our study is in line with the study of Ez-Zaitouni et al. in two European cohorts because they reported that a PFH of AS is associated with HLA-B27 carriership, but a PFH of ReA, IBD, or psoriasis was not [14]. In this study, a PFH of ReA, IBD, and AAU was also less common than a PHF of AS. However, in contrast to our findings, Ez-Zaitouni et al. [14] also found that a PFH of AAU contributed to the identification of axSpA because this was associated with HLA-B27 carriership in patients with CBP. In our study, a PFH of AAU was rarely reported (1%), as compared with 5–6% in the study of Ez-Zaitouni et al., which may limit detecting a possible association with HLA-B27 carriership owing to sample size. We have no proper explanation why a PFH of AAU was less common in our study. It may be attributed to differences in ethnicity (European cohorts vs. worldwide cohort in our study). However, the frequency of a PFH of AAU was similarly low in white and Asian patients in our cohort (0.7% vs. 0.5%). Moreover, the frequency of AAU in patients suspected of axSpA was similar in our cohort (9% overall; 10% in white and 6% in Asian patients) as compared with 8% in the study by Ez-Zaitouni et al.

In a general practice setting or other low SpA prevalence settings, a PFH of AS could be used for identifying HLA-B27-positive patients among patients suspected of axSpA. The RADAR study showed that HLA-B27 is a good referral tool for identifying patients with axSpA, because HLA-B27 has a higher sensitivity than an ASAS PFH [17]. Therefore, it is recommended that a PFH of AS should be used as a criterion for referral to secondary care only when HLA-B27 testing is not feasible.

The current study investigated patients with axial symptoms (i.e., patients with CBP suspected of axSpA) of the ASAS cohort, and therefore it is important to emphasize that the results are not applicable to patients with predominantly peripheral symptoms. In patients with predominantly peripheral symptoms, a PFH of, for example, psoriasis could be important and relevant for identifying patients with an increased risk of pSpA [15].

An important strength of this study is that the ASAS study is a worldwide cohort, which enabled us to investigate different self-reported ethnicities. Another strength is the availability of extensive information on family history, which allowed us to investigate the role of first- and second-degree relatives for each manifestation of a PFH. A major limitation is the self-reported family history by patients. This could lead either to an underestimation if a patient forgets or is unaware that a relative has an SpA-related disease or to an overestimation if a patient is confused or mistaken in the type of disease of a relative. Nevertheless, in a clinical setting, the physician usually has to depend on patient-reported family history. Another limitation is the small percentage of patients with self-reported ethnicity other than being white or Asian. Only 5% of the patients reported to be Hispanic/Latino, black, mixed, Turkish, East Indian, or unknown ethnicity. Therefore, the results are applicable only to white or Asian populations, which corresponds to the largest population with axSpA worldwide [18]. Preferably, future research into the value of a PFH for identifying HLA-B27 carriership should be conducted in patients with other ethnicities. In this study, we focused on the use of a PFH of SpA for identifying patients with CBP who are at an increased risk of HLA-B27 carriership, but we did not investigate the potential added value of a PFH in diagnosing patients with axSpA. We are currently analyzing data from three independent axSpA cohorts to address this issue.

## Conclusions

Our data, in combination with data from two European cohorts, show that a PFH of AS and possibly also a PFH of AAU, regardless of ethnicity (white or Asian) or degree of family relationship, is valuable for identifying patients with CBP who could be HLA-B27-positive and consequently have an increased risk of axSpA.

## Abbreviations

AAU: Acute anterior uveitis
AS: Ankylosing spondylitis
ASAS: Assessment of SpondyloArthritis international Society
axSpA: Axial spondyloarthritis
CBP: Chronic back pain
CRP: C-reactive protein
ESR: Erythrocyte sedimentation rate
FDR: First-degree relative
HLA-B27: Human leukocyte antigen B27
IBD: Inflammatory bowel disease
IBP: Inflammatory back pain
MRI: Magnetic resonance imaging
NSAID: Nonsteroidal anti-inflammatory drug
PFH: Positive family history
pSpA: Peripheral spondyloarthritis
ReA: Reactive arthritis
SDR: Second-degree relative
SI: Sacroiliac joints
SpA: Spondyloarthritis
